# Impaired autophagosome clearance contributes to neuronal death in a piglet model of neonatal hypoxic-ischemic encephalopathy

**DOI:** 10.1038/cddis.2017.318

**Published:** 2017-07-13

**Authors:** Derong Cui, Dawei Sun, Xintao Wang, Liye Yi, Ewa Kulikowicz, Michael Reyes, Junchao Zhu, Zeng-Jin Yang, Wei Jiang, Raymond C Koehler

**Affiliations:** 1 Department of Anesthesiology, Shanghai Sixth People's Hospital Affiliated with Shanghai Jiao Tong University, No. 600, Yi Shan RD, Shanghai 200233, China; 2 Department of Anesthesiology and Critical Care Medicine, Johns Hopkins University, Baltimore, MD21287, USA; 3 Department of Neurosurgery, The Second Affiliated Hospital of Harbin Medical University, No.148, Bao Jian RD., Harbin 150086, China; 4 Department of Anesthesiology and Critical Care Medicine, Johns Hopkins University, 1800 Orleans St., Baltimore, MD 21287, USA; 5 Department of Anesthesiology, Shengjing Hospital of China Medical University, No.36, San Hao St., Shenyang 110004, China

## Abstract

To examine the temporal relationship of cortical autophagic flux with delayed neuronal cell death after hypoxia-ischemia (HI) in neonatal piglets. HI was produced with 45-min hypoxia and 7-min airway occlusion in 3–5-day-old piglets. Markers of autophagic, lysosomal and cell death signaling were studied via immunohistochemistry, immunoblotting, and histochemistry in piglet brains. *In vitro*, autophagy was impaired in cultured mouse cortical neurons treated with chloroquine with or without rapamycin for 1 d in the presence of Z-VAD-fmk, cyclosporine A, or vehicle control, and cell viability was assessed with the MTT assay. *In vivo*, neuronal cell death of sensorimotor cortex was delayed by 1–2 days after HI, whereas LC3-II, Beclin-1, PI3KC3, ATG12-ATG-5, and p-ULK1 increased by 1.5–6 h. Autophagosomes accumulated in cortical neurons by 1 d owing to enhanced autophagy and later to decreased autophagosome clearance, as indicated by LC3, Beclin-1, and p62 accumulation. Autophagy flux impairment was attributable to lysosomal dysfunction, as indicated by low lysosomal-associated membrane protein 2, cathepsin B, and cathepsin D levels at 1 d. Ubiquitin levels increased at 1 d. Autophagosome and p62 accumulated predominantly in neurons at 1 d, with p62 puncta occurring in affected cells. Beclin-1 colocalized with markers of caspase-dependent and caspase-independent apoptosis and necrosis in neurons. *In vitro*, mouse neonatal cortical neurons treated with rapamycin and chloroquine showed increased autophagosomes, but not autolysosomes, and increased cell death that was attenuated by cyclosporine A. Neonatal HI initially increases autophagy but later impairs autophagosome clearance, coinciding with delayed cortical neuronal death.

Autophagy can be divided into macro-, micro-, and chaperone-mediated autophagy. Macroautophagy (hereafter referred to as simply ‘autophagy’) helps in cellular homeostasis by mediating the bulk degradation and recycling of cytoplasmic organelles. This evolutionarily conserved, catabolic process is initiated by crescent-shaped structures called phagophores. Phagophores enclose cytosolic components, and then expand and fuse together to form closed, double-membrane vesicles termed autophagosomes. The autophagosomes are, in turn, targeted by lysosomes/vacuoles, which fuse with them and degrade their cytosolic contents.^1, 2^

Basal levels of autophagy are essential for the degradation of abnormal proteins and organelles. Under conditions of stress, autophagic activity is increased, and this may enable cells to survive the stressor or lead to cell death.^3, 4, 5^ Studies on rodent models of perinatal hypoxia-ischemia (HI) have indicated that cell death occurs along a ‘continuum’ of apoptosis to necrosis, and features of both these processes can be detected in varying proportions in different pathologies.^6, 7^ Moreover, in addition to these cell death mechanisms, enhanced autophagy has been detected in dying neurons after HI injury following asphyxial cardiac arrest (CA).^8, 9, 10^ However, the role of autophagy in HI-mediated neuronal death is not fully understood, and the specific cell type(s) involved and the underlying mechanisms remain unclear. Recent studies indicate that autophagy can serve as a trigger of apoptosis or necrosis, or act as an independent mechanism of cell death.^11, 12^ Moreover, both beneficial and detrimental effects of autophagy have been reported after neonatal HI.^8, 9, 11, 13^

In the present study, we determined the levels of autophagy and autophagic flux after neonatal HI induced by asphyxial CA in newborn piglets, as the brain regions vulnerable to HI in neonatal piglets correspond to those in human term newborns.^14, 15, 16^ We postulated that increased autophagy flux is an early event in this region, but that later lysosomal dysfunction impairs autophagosome clearance that precedes or coincides with delayed neuronal cell death signaling. We also directly tested the effect of impaired autophagosme clearance in neurons *in vitro*.

## Results

### Autophagosome accumulation in the brain after HI

**Figure 1:**
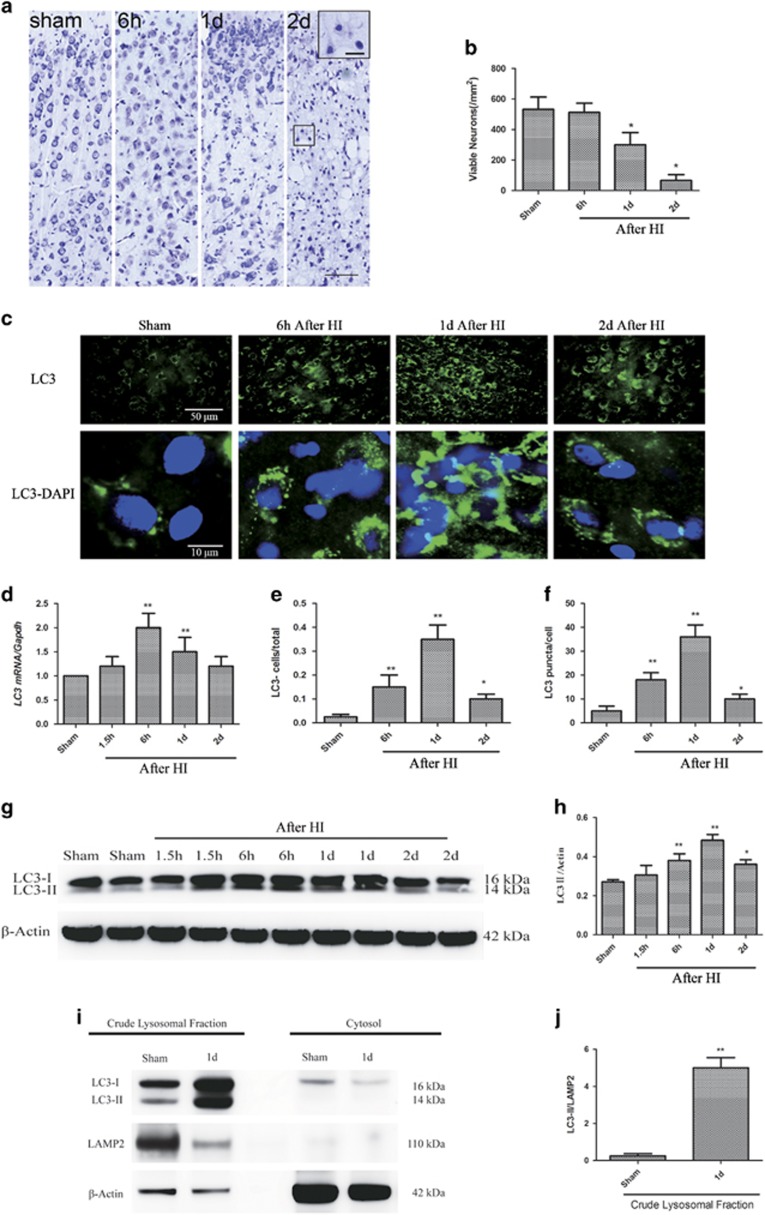
Autophagosomes accumulate in the cortex at 1 d after HI. (**a**) HI is associated with severe neurodegeneration from 1 d to 2 d of recovery. Paraformaldehyde-fixed brain sections are stained with cresyl violet. The images show the morphology of viable neurons in the cortex of sham-operated and HI piglets at 6 h, 1 d, and 2 d after HI. Scale bar=50 *μ*m at low power and 10 *μ*m in high power inset. (**b**) Quantification of viable cortical neurons in sham-operated and HI piglets (n=3, *P<0.05 at 1 d and 2 d after HI as compared with the sham-operated group). (**c**) Images of the cortical LC3 signal in sham-operated and HI piglets. The puncta correspond to phagophores and/or autophagosomes (arrowheads). The nuclei are stained with DAPI. Scale bar=50 *μ*m for low power and 10 *μ*m for high power images. (**d**) Relative LC3 mRNA levels (qPCR) in the cortices of sham-operated and HI piglets, normalized to the experimental control, GAPDH (*n*=3, **P<0.01 at 6 h and 1 d after HI, as compared with the sham-operated group). (**e**) Quantification of cortical LC3 signals in sham-operated and HI piglets, normalized to the total cell number (*n*=3, *P<0.05, **P<0.01 *versus* the sham-operated group). (**f**) Quantification of LC3 puncta data from (E) normalized to the total cell number. (*n*=3, *P<0.05 *versus* the sham-operated group). (**g**) Western blots of LC3 in cortical tissue lysates from sham-operated and HI piglets at the indicated time points. Each lane corresponds to an individual animal (2 per time point). (**h**) Densitometric analysis of LC3-II data from (**g**) normalized to the loading control, *β*-actin (*n*=4, *P<0.05, **P<0.01 *versus* the sham-operated group). (**i**, **j**) Autophagosomes accumulate in the crude lysosomal fraction (CLF) of the cortex after HI. (**i**) Representative images of western blot analyses of subcellular fractions (CLF and cytosol) of the cortices of sham-operated and HI piglets at 1 d after the injury for the autophagosomal marker LC3, lysosomal marker LAMP2, and cytosolic marker *β*-actin. (**j**) Densitometric analysis of LC3-II in CLFs of cortices of sham-operated and HI piglets. (*n*=4, **P<0.01, *versus* the sham-operated group). All data are presented as mean±S.D.

The morphology of viable neurons in the sensorimotor cortex of sham-operated and HI piglets was examined on cresyl violet-stained sections (Figure 1a). Whole-body HI following asphyxial CA was associated with severe and progressive neurodegeneration from 1 d to 2 d after HI (*P*<0.05, Figure 1b).

Next, we determined the levels of LC3 (microtubule-associated protein 1 light chain 3), an autophagy marker, in the vulnerable sensorimotor cortex at 1.5 h, 6 h, 1 d, and 2 d after HI. Within 6 h after HI, the levels of LC3 mRNA and the number of LC3-positive cells were significantly higher in the cortices of the injured animals than in those of the uninjured sham controls (*P*<0.01, Figures 1c, d, and h), suggesting that HI induced autophagy. The numbers of LC3-positive cells peaked at 6 h and 1 d (*P*<0.01) after HI and then subsided but still remained elevated at 2 d (*P*<0.05). Examination at high magnification revealed the accumulation of punctate LC3-positive autophagic structures in the injured cortex (*P*<0.01, Figures 1d and i). Phosphatidylethanolamine converts LC3-I to LC3-II and results in autophagosome formation.^17^ We therefore performed Western blot analysis of LC3-II, as a marker of autophagy, after the addition of phosphatidylethanolamine to the sections. LC3-II levels increased in a time-dependent manner, peaking between 6 h and 1 d after HI (*P*<0.01) and then gradually decreasing by 2 d (*P*<0.05, Figures 1g and h). In addition, we detected LC3-II accumulation in the crude lysosomal/membrane fraction but not in the cytosolic fraction prepared from the cortices of the injured piglets as compared with the sham-operated piglets (*P*<0.01, Figures 1i and j). This finding confirmed that lipidated LC3 associated with membranes in the brain cells after HI.

**Figure 2:**
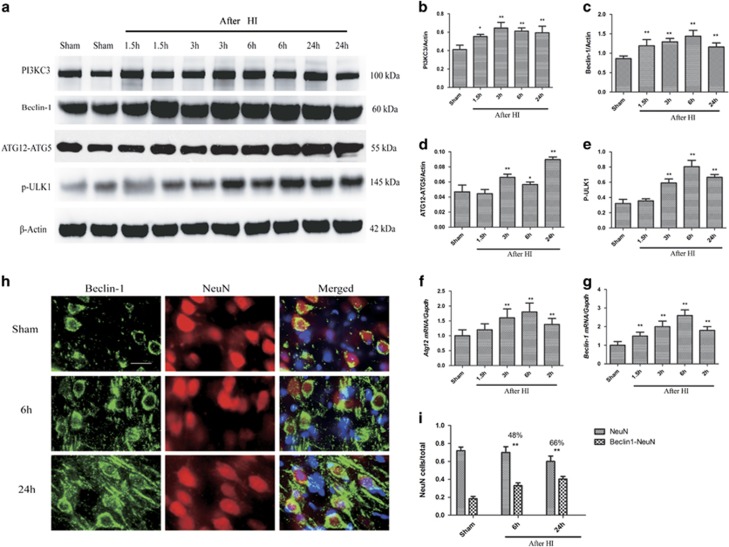
Autophagosomes accumulate in the cortex from 1.5 h to 1 d after HI. (**a**) Western blots of PIK3C3/VPS34, Beclin-1, ATG12-ATG5 conjugate, and phospho-ULK1 in cortical tissue lysates from sham-operated and HI piglets at the indicated time points. Each lane corresponds to an individual animal (two per time point). (**b**–**e**) Densitometric analysis of (**b**) PIK3C3/VPS34, (**c**) Beclin-1, (**d**) ATG12-ATG5 conjugate, and (**e**) phospho-ULK1 data from (A) normalized to the loading control, *β*-actin (*n*=4, *P<0.05, **P<0.01 *versus* the sham-operated group). (**f**–**g**). Quantitative PCR results of Atg12 mRNA (F) and Beclin-1 mRNA (G). (**h**) Images of piglet cortical brain sections stained with antibodies against the autophagy marker Beclin-1 and the neuronal marker NeuN. Scale bar=50 *μ*m. (**i**) Quantification of NeuN-positive cells and Beclin-1 and NeuN double-positive cells normalized to the total cell number (**P<0.01 *versus* the sham-operated group). The percentages of double-positive *versus* single-positive cells are indicated at 6 h and 1 d after HI. Data are presented as mean±S.D. (*n*=3; at least 1000 cells were quantified per piglet per experiment)

The PIK3C3/VPS34–Beclin-1 complex and the ULK1 complex contribute to autophagy regulation and initiation, whereas ATG12-ATG5 conjugates participate in phagophore elongation.^18^ We found that the levels of PIK3C3, Beclin-1, ATG12-ATG5 conjugate, and phospho-ULK1 in the sensorimotor cortex were significantly higher in the injured piglets than in the sham-operated controls (*P*<0.01, Figures 2a–e). The ATG12-ATG5 conjugate level and the Beclin-1 and Atg12 mRNA levels gradually increased between 1.5 h and 24 h (*P*<0.01, Figures 2f and g). These findings indicate that autophagy initiation was increased soon after cerebral HI. Considering the increase in downstream mediators, it is likely that enhanced autophagy induction led to increased autophagosome formation, and may partly account for the observed LC3-II accumulation. Collectively, the data show that LC3 and phagophores/autophagosomes accumulated in the vulnerable sensorimotor cortex prior to morphologically identified cell death. We also found significant colocalization of Beclin-1 (an autophagy marker) with NeuN (a neuronal marker) in the cortex at 1 d following HI (*P*<0.01, Figures 2h and i), indicating that phagophore/autophagosome accumulation occurred specifically in neurons.

### Effect of HI on the brain autophagic signaling pathway

To investigate the effect of HI following asphyxial CA on the brain autophagic signaling pathway, we measured the levels of phospho-mTOR and phospho-p70S6K, a representative downstream target of mTOR,^19^ in brain homogenates.^20^ We found that the level of phospho-mTOR decreased significantly by 1.5 h of reoxygenation from HI (*P*<0.01, Supplementary Figure S1A and B) and that the level of phospho-p70S6K decreased significantly by 6 h (*P*<0.01, Supplementary Figure S1A and C), thereby supporting involvement of the mTOR pathway in the autophagic response in postischemic brain.

### Autophagosome accumulation in neurons, microglia, and astrocytes after HI

**Figure 3:**
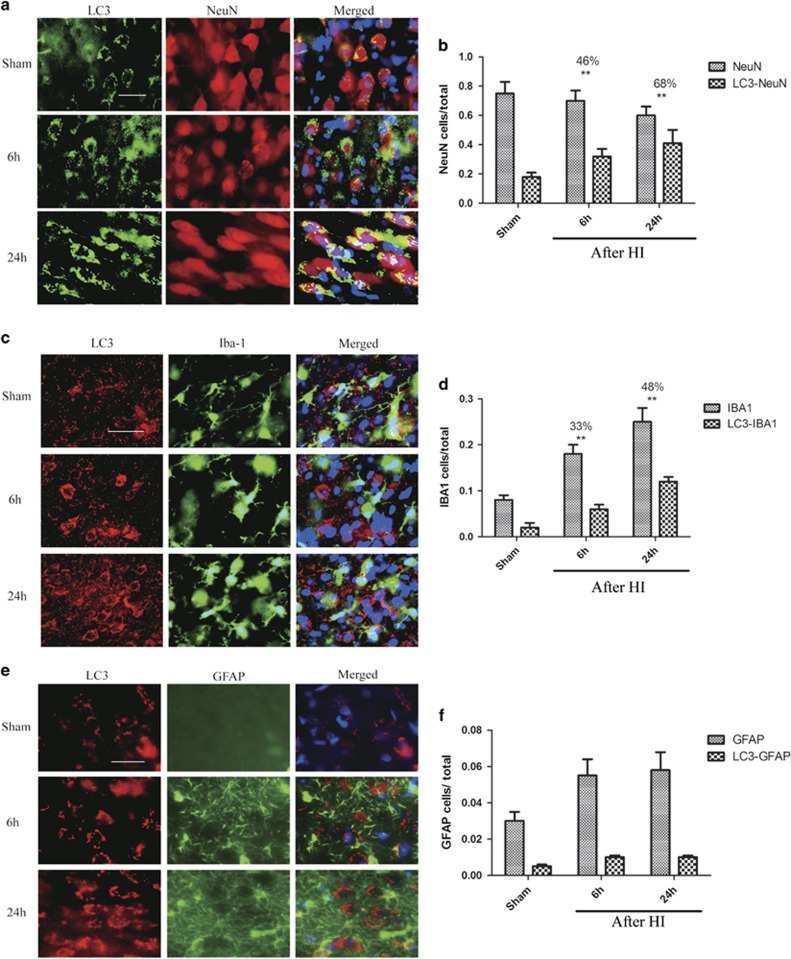
Autophagosome accumulation in cortex after HI is cell type specific. Images of cortical brain sections stained with antibodies against LC3 and (**a**) the neuronal marker NeuN, (**c**) the microglial marker IBA1, and (**e**) the astrocyte marker GFAP. Merged images also include DAPI nuclear staining (blue). Scale bar=50 *μ*m. Quantification of cells positive for each marker and cells double-positive for LC3 along with (B) NeuN (**P<0.01), (**d**) IBA1 and (**e**) GFAP, normalized to the total cell number. The percentages of LC3-NeuN double-positive cells *versus* single-positive cells are indicated at 1 d. Data are presented as mean±S.D. (*n*=3 piglets; at least 1000 cells were quantified per piglet per experiment)

We observed significant LC3 and NeuN colocalization at 1 d after HI (*P*<0.01, Figures 3a and b), indicating that phagophore/autophagosome accumulation occurred specifically in neurons. At this time point, 48% of cells that expressed IBA1, a microglia marker, were positive for LC3 (*P*<0.01, Figures 3c and d). Although IBA1 is expressed by both ramified and amoeboid microglia, the LC3 signal was mainly localized in amoeboid microglia in the injured brain area. This suggested that phagophores and/or autophagosomes accumulated in activated microglia. Colocalization of LC3 and GFAP (Figures 3e and f) was sparse at all of the examined time points, indicating that increased autophagy was not prominent in astrocytes after HI (*P*>0.05).

In addition to sensorimotor cortex, we found significant LC3-NeuN colocalization in putamen, thalamus, and hippocampus at 1 d after HI (Supplementary Figure S2). These regions are also selectively vulnerable to HI.^21^ Poor LC3-NeuN colocalization was seen in the subcortical white matter.

### Autophagosome accumulation after HI is partly attributable to impaired autophagy flux

**Figure 4:**
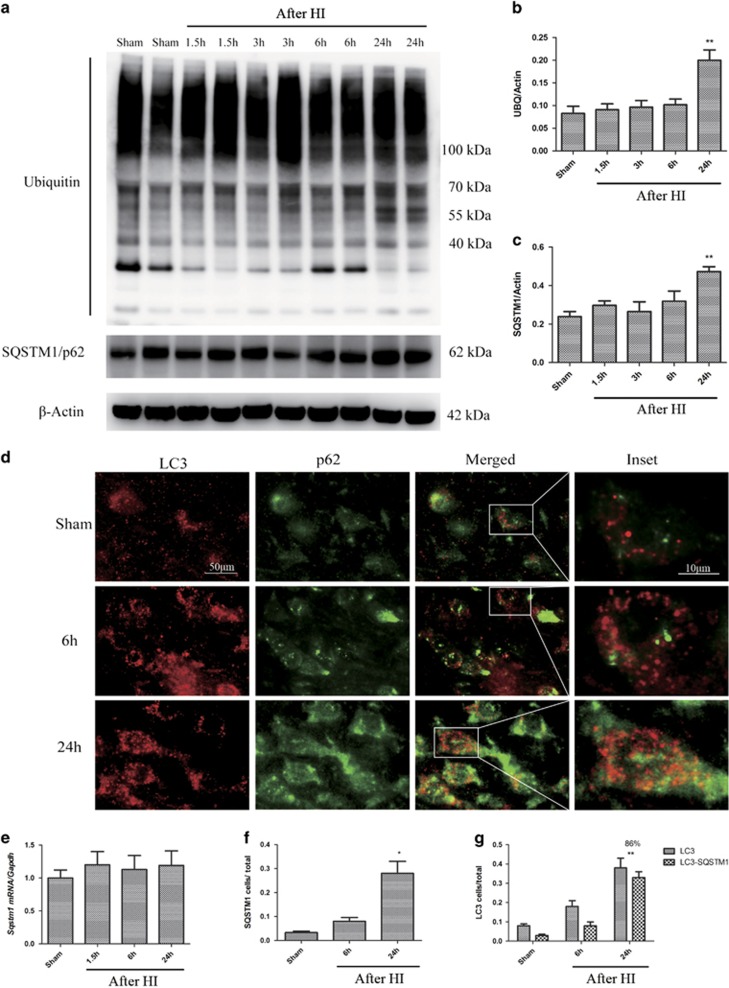
Autophagic turnover in the cortex is impaired after HI. (**a**) Western blot analysis of ubiquitin (UBQ) and SQSTM1/p62 in cortical tissue lysates from sham-operated and injured animals. (**b**, **c**) Densitometric analysis of (**b**) total ubiquitinated proteins and (**c**) SQSTM1/p62 with respect to the loading control, *β*-actin (*n*=4, **P<0.01). (**d**) Images of sensorimotor cortical sections stained with antibodies against LC3 and SQSTM1/p62. Scale bar=50 *μ*m for low power images and 10 *μ*m for high power insets. (**e**) Relative mRNA level of SQSTM1/p62 in the cortices of uninjured control and injured piglets (*n*=3). (**f**) Quantification of immunofluorescence data showing the number of SQSTM1/p62-positive cells in cortical brain sections from sham-operated and HI piglets (*n*=3, *P<0.05 at 1 d after HI). (**g**) Quantification of LC3-positive cells, and LC3 and SQSTM1/p62 double-positive cells. The percentages of double-positive *versus* single-positive cells are indicated (*n*=3, **P<0.01 at 1 d after HI; at least 1000 cells were quantified per piglet per experiment)

Ubiquitinated cargo, including injured organelles and potentially toxic protein aggregates, are delivered to autophagosomes by the receptor protein SQSTM1/p62.^22^ Therefore, stimulation of the autophagy flux will deplete p62 and other autophagic substrates, whereas impaired autophagic clearance will lead to p62 accumulation within cells.^23^ We found that p62 protein levels markedly increased in the vulnerable sensorimotor cortex within 1 d after HI (*P*<0.01, Figures 4a and c), whereas no significant changes in p62 mRNA levels were apparent at this time (*P*>0.05, Figure 4e). These observations are consistent with autophagic protein degradation being increased immediately after HI but then being impaired at later time points. Consistent with this defect in protein degradation, we observed the accumulation of ubiquitinated proteins (Figures 4a and b). Similar to p62, ubiquitinated protein levels gradually increased at 1 d after HI (*P*<0.01). Because these proteins are degraded by the proteasome, the observed increase in their level may be attributable to impaired proteasomal degradation after HI^24^ or to insufficient time after HI to clear all accumulated autophagic cargo. Consistent with the western blot data, the immunohistochemical data showed markedly higher p62 levels in the damaged cortex as compared with the uninjured sham controls (*P*<0.05, Figures 4d and f). In addition, strong p62-LC3 colocalization was observed 1 d after HI (*P*<0.01, Figures 4d and g). Furthermore, cortical LC3 accumulation peaked at 1 d (*P*<0.01) and then decreased by 2 d after HI (*P*<0.05, Figures 1d–h). Collectively, these findings suggest that autophagosome clearance was partially impaired in the cortex, leading to the accumulation of ubiquitinated proteins and protein aggregates, which can contribute to neuronal death.^25, 26^ Consistent with this possibility, we observed an increased ubiquitin signal and severe neurodegeneration at 1 d after HI (*P*<0.01), suggesting that impaired autophagy flux led to ubiquitinated protein accumulation paralleling the onset of neuronal death.

### Lysosomal malfunction leads to autophagy disruption after HI

**Figure 5:**
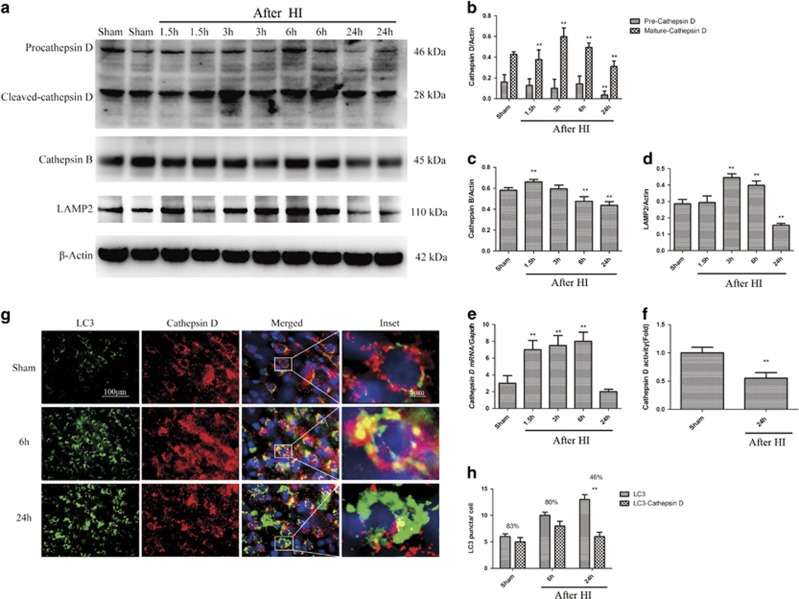
HI leads to lysosomal dysfunction. (**a**) Western blot analysis of cathepsin D, cathepsin B, and LAMP2 in cortical tissue lysates from sham-operated and HI piglets. Densitometric analysis of precursor and mature forms of cathepsin D (**b**), cathepsin B (**c**), and LAMP2 (**d**) with respect to the loading control *β*-actin (*n*=4, **P<0.01). (**e**) Relative mRNA level (qPCR) of cathepsin D in the cortices of uninjured control and injured piglets, normalized to the loading control, GAPDH (*n*=3, **P<0.01 *versus* the sham-operated group). (**f**) Cathepsin D enzyme activity determined by *in vitro* fluorometric assay in the crude lysosomal fraction prepared from the cortices of sham-operated and injured piglets (*n*=5, **P<0.01). (**g**) High magnification images of cortical cells stained with antibodies against LC3 and cathepsin D. Accumulation of LC3 and cathepsin D double-positive structures (arrowheads) and depletion of single cathepsin D-positive lysosomes (arrows) is apparent after HI. Scale bar=50 *μ*m for low power and 10 *μ*m for high power inset. (H) Quantification of LC3 puncta and LC3/cathepsin D double-positive puncta in the cortices of sham-operated and HI piglets. The percentage of overlap is indicated (*n*=3, **P<0.01; data are presented as mean±S.D.)

Autophagosomes are degraded by lysosomal hydrolases.^1, 27, 28^ To determine whether HI disrupted lysosomal integrity and led to lysosomal enzyme leakage into the cytosol, we prepared crude lysosomal and cytosolic fractions from the cortices of injured and sham-operated piglets, and measured the levels of lysosomal-associated membrane protein 2 (LAMP2), a soluble lysosomal enzyme, in both fractions by using protein gel blots. In both sham-operated and injured animals, LAMP2 was detected in the lysosomal fraction and only faintly detected in the cytosolic fraction (*P*<0.01, Figures 1i and j). This result indicates that lysosomal integrity likely was maintained after HI. We also measured the levels of lysosomal proteins in total protein lysates of the cortex by using western blot analysis. The cortical level of cleaved-cathepsin D (28 kDa) was markedly increased from 1.5 to 6 h after HI (*P*<0.05). By 1 d, however, this level was slightly lower in the injured animals than in the sham-operated controls (*P*<0.01, Figures 5a and b). Cathepsin D mRNA levels in the cortex were slightly elevated from 1.5 to 6 h after HI (*P*<0.01) and significantly decreased at 1 d (*P*<0.01). Similar expression patterns were found for the lysosomal membrane proteins cathepsin B and LAMP2 (*P*<0.01, Figures 5a, c, and d). To determine whether the decreased cathepsin D levels impaired lysosomal function and thereby impaired autophagic degradation, we analyzed the enzymatic activity of cathepsin D in cortical extracts. This activity was significantly lower in the injured animals than in the controls at 1 d after HI (*P*<0.01, Figure 5f), consistent with impaired lysosomal function. Because lysosomal function is required for autophagosome-lysosome fusion, the above finding may account for the decreased autophagosome clearance observed in this study. To confirm this possibility, we evaluated the colocalization of lysosomes and autophagosomes after HI. High-resolution analysis of brain sections showed decreased colocalization of cathepsin D-positive lysosomes with LC3-positive autophagosomes in the injured animals as compared with the sham-operated animals (*P*<0.01, Figures 5g and h). In addition, few unfused lysosomes were observed in the cortex in the injured animals as compared with the sham-operated animals. This observation is consistent with the hypothesis that the decrease in cathepsin D protein level and activity after HI led to the accumulation of unfused or partially fused autophagosomes that could not be efficiently cleared by the lysosomes.

### Impaired autophagosome clearance in association with neuronal death signaling

Neonatal HI following asphyxial CA was associated with severe neurodegeneration as observed on cresyl violet staining (*P*<0.05, Figures 1a and b). The cortical levels of cleaved caspase-3, cleaved poly(ADP-ribose) polymerase (PARP), and spectrin breakdown products increased after HI, peaking at 1 d after HI (*P*<0.01, Supplementary Figure S3A–D). These observations indicate that caspase- and calpain-dependent signaling were active in the injured cortex at the time of maximal autophagy impairment. Some authors have reported that autophagy may act as a mechanism for programmed cell death.^29^

To verify that impaired autophagosome clearance can lead to cell death in neurons, we treated cultured cortical neurons from neonatal mice with rapamycin, which is an irreversible mTOR inhibitor.^30^ Rapamycin treatment increases autophagic activity by increasing autophagic flux. The neurons were also subjected to nutrient deprivation, which also stimulates autophagy. To block autophagosome clearance, we treated neurons with chloroquine, which increases lysosomal pH and prevents autophagosome-lysosome fusion, thereby inhibiting lysosomal function.^31, 32^ We evaluated the autophagic flux by assessing LC3-II and p62 expression in the presence and absence of chloroquine.

**Figure 6:**
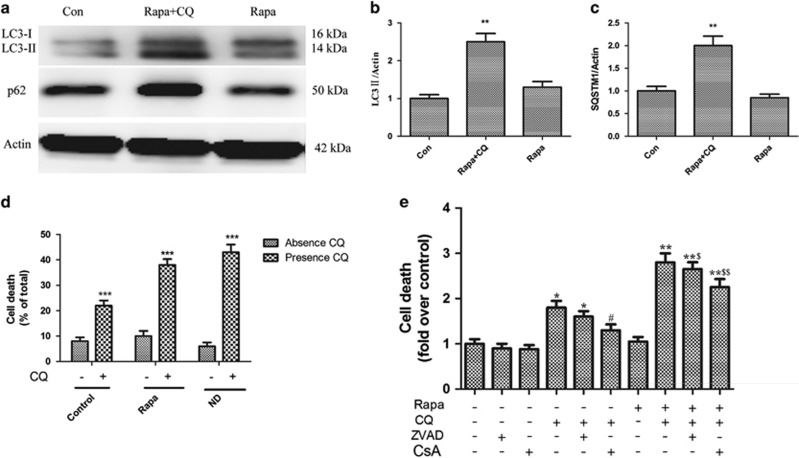
Autophagy impairment contributes to cell death in cultured neurons. (**a**) Western blot analysis of LC3-I/II and p62 in primary cultured neurons treated with chloroquine (5 *μ*mol/l) with or without rapamycin (100 nmol/l) treatment for 1 d. (**b**, **c**) Corresponding densitometric analysis of (**b**) LC3-I/II and (**c**) p62 bands with respect to *β*-actin (*n*=4, **P<0.01). (**d**) Cell death in primary cultured neonatal cortical neurons treated with chloroquine (5 *μ*mol/l) with or without rapamycin (100 nmol/l) treatment and nutrient deprivation (ND) for 1 d (***P<0.001 *versus* the non-chloroquine treatment group). (**e**) Cell death in primary cultured neurons treated with chloroquine (5 *μ*mol/l) with or without rapamycin (100 nmol/l) treatment for 1 d in the presence of Z-VAD-fmk (10 *μ*mol/l), cyclosporine A (CsA; 10 *μ*mol/l), or DMSO (control). *P<0.05, *P<0.01, *versus* control, #*P*<0.05 *versus* the chloroquine treatment group, $*P*<0.05, $$*P*<0.01 *versus* the rapamycin plus chloroquine treatment group

Rapamycin treatment alone did not significantly change LC3-II or p62 levels (*P*>0.05, Figures 6a–c), suggesting that changes in autophagic flux were balanced by changes in lysosomal clearance. However, chloroquine pretreatment before rapamycin increased autophagosome-bound LC3-II and p62 (*P*<0.01, Figures 6a–c). This result indicates that chloroquine produced the expected impairment of autophagosome clearance. Importantly, chloroquine treatment was sufficient to cause neuronal cell death, as assessed by the MTT assay, in both the basal state and under the conditions of rapamycin and nutrient deprivation–stimulated autophagy (*P*<0.001, Figures 6d). In other cell types, chloroquine-induced autophagosome accumulation is known to prevent the clearance of damaged intracellular organelles and proteins, and to lead to increased reactive oxygen species (ROS) generation with loss of mitochondrial membrane potential, causing mitochondrial permeabilization and activating programmed apoptosis and/or necrosis.^29^ Furthermore, chloroquine-induced ROS generation was found to persist after pretreatment with cyclosporine A, an inhibitor of mitochondrial permeability transition pore, thereby indicating that ROS generation occurs upstream of the mitochondrial permeabilization. In our study in neurons, pretreatment with cyclosporine A but not Z-VAD-fmk (a pan-caspase inhibitor) significantly attenuated chloroquine-induced cell death under conditions of both basal and rapamycin-stimulated autophagy (*P*<0.01, Figures 6e). These findings suggest that autophagosome accumulation led to neuronal cell death but that caspase activity was not required for cell death execution.

### Impaired autophagy leads to neuronal death after HI *in vivo*

To further examine if the inhibition of autophagy flux contributed to cell death after HI *in vivo*, we measured the levels of markers of different types of cell death signaling, and determined whether these markers colocalized with the autophagy marker Beclin-1. The cortical levels of cleaved caspase-3, cleaved caspase-9, and cleaved PARP, a product of caspase activity,^33^ were increased after HI with the peaks occurring at 1 d (*P*<0.01, Supplementary Figure S3A–D and S4A and B). Cleaved caspase-9 strongly colocalized with Beclin-1, which was predominantly localized in the neurons, at 1 d after HI (*P*<0.01, Figures 2h and i). At the same time point, the neuronal autophagic flux was impaired. Thus, impairment of autophagic flux temporally correlated with markers of caspase activation *in vivo*.

An increase in the number of AIF-positive cells, 50% of which colocalized with Beclin-1, was detected at 1 d after HI (*P*<0.01, Supplementary Figure S4C and D). Because neurons were the major cell type showing autophagosome accumulation at 1 d of recovery, the above finding suggests that the blockage of autophagic clearance also temporally correlates with the caspase-independent AIF signaling pathway. Thus, autophagic impairment may contribute to both caspase-dependent and caspase-independent neuronal cell death signaling after HI.

Endoplasmic reticulum (ER) stress has been reported to be involved in autophagy and apoptosis after cerebral ischemia/reperfusion.^34^ We evaluated the expression of caspase-12, which can be associated with ER stress. Caspase-12 levels were increased and significant caspase-12 and Beclin-1 colocalization was observed in the cortex at 1 d after HI (*P*<0.01, Supplementary Figure S4E and F). Moreover, the majority of caspase-12 and Beclin-1 double-positive cells exhibited neuronal morphology. These findings suggest that impaired autophagy is associated with ER stress in neurons.

Finally, we examined whether autophagy was impaired in necrotic cells. In neonatal HI encephalopathy, neurons undergo apoptosis and necrosis;^35, 36^ However, impairment of protein synthesis and mitochondrial dysfunction result in the interruption of apoptosis and lead to neuronal necrosis.^7, 37, 38^ In our previous HI piglet model, neurons in the sensorimotor cortex morphologically appeared predominantly necrotic rather than apoptotic at 2 d after HI.^15^ One form of programmed necrosis is dependent on the receptor-interacting protein 1 (RIP1) serine-threonine kinase activity acting on RIP3 and on the subsequent RIP1 and RIP3 interaction with mixed-lineage kinase domain-like protein (MLKL) scaffolding protein. RIP1- and RIP3-containing protein complexes that form specifically in response to necrosis have been identified. One complex contains the mitochondrial protein phosphatase PGAM5, which has two splice variants: PGAM5L (long form) and PGAM5S (short form).^39^ Upon induction of RIP1-dependent necrosis, PGAM5S recruits Drp1, a mitochondrial fission factor, and activates its GTPase activity via dephosphorylation of its serine 637 site. Drp1 activation results in mitochondrial fragmentation. We found increased protein expression of RIP1, RIP3, MLKL, and PGAM5 in the cortex, and these levels peaked at 1 d after HI. Immunostaining of the cortical sections revealed an increase in RIP3-positive cells (*P*<0.01, Supplementary Figure S5A and B), MLKL-positive cells (*P*<0.01, Supplementary Figure S5C and D), and PGAM5-positive cells (*P*<0.01, Supplementary Figure S5E and F) at 1 d after HI. Colocalization with Beclin-1 was present in 85% of cells positive for RIP3, 72% of cells positive for MLKL, and 80% of cells positive for PGAM5. Because neurons were the predominant cell type showing autophagosome accumulation, the above findings suggest that the blockage of autophagic clearance also temporally correlates with neuronal RIP1-dependent necrosis. Collectively, our data suggest that autophagic impairment may contribute to increases in caspase-dependent apoptotic signaling and to caspase-independent cell death signaling involving the AIF and RIP1-dependent pathways after HI.

## Discussion

Autophagic markers have been shown to be increased in the brain after neonatal HI, but the underlying mechanisms are unknown.^10^ We found that upstream regulators and mediators of autophagy were increased as early as 1.5–3 h after neonatal HI, leading to increased autophagy induction, which partially accounted for autophagosome accumulation in the early stages after HI. However, we also observed the accumulation of autophagy substrates and impairment of autophagy flux mainly within neurons and partly within activated microglia at 1 d after neonatal HI. These findings were attributed, at least in part, to lysosomal dysfunction. In lysosomal storage diseases, defects in specific lysosomal hydrolases lead to lysosomal dysfunction and consequently inhibit autophagy.^40, 41^ Lysosomal dysfunction has also been detected in some neurodegenerative diseases and traumatic brain injury.^42, 43^ In the present study, we demonstrated that lysosomal dysfunction occurred at later time points after neonatal HI following asphyxial CA, and contributed to defective autophagic clearance, which in turn, was temporally related to multiple neuronal cell death signaling pathways and morphologically identified cell death.

Caspase-9 is involved in the intrinsic mitochondrial death pathway.^44^ Caspase-9 cleaves and activates downstream effector caspases, such as caspase-3, which can eventually lead to endonuclease degradation of DNA. We detected the colocalization of cleaved caspase-9 with Beclin-1, which suggested that impaired autophagic clearance was associated with activation of caspase signaling at 1 d after HI.

In addition, we detected the colocalization of AIF with Beclin-1. AIF mediates PARP-dependent cell death that is independent of caspase signaling. During over-activation of PARP caused by ROS-induced damage to DNA, AIF is translocated from the mitochondrial membrane to the cytosol and eventually to the nucleus, where it has a role in endonuclease activation.^45^ One explanation for the observed AIF and Beclin-1 colocalization at 1 day after HI is that impaired autophagic clearance led to the accumulation of damaged mitochondria that may have facilitated access of PARP-generated poly(ADP-ribose) polymers to a larger pool of AIF and enhanced release of AIF. In addition to PARP-dependent nuclear translocation of AIF, other forms of programmed necrosis have been described in neurons. One prominent form in stroke is RIP1-dependent necrosis, which involves the interaction of RIP1 with RIP3, the docking protein MLKL, and possibly the mitochondrial protein PGAM5.^39^ Here, we found a marked increase in the colocalization of Beclin-1 with RIP3, MLKL, and PGAM5. Thus, it appears that multiple necrotic cell death signaling pathways can be recruited during impaired autophagosome clearance. Interestingly, our *in vitro* results supported a caspase-independent pathway. Neuronal cell death following chloroquine-induced impairment of autophagic clearance did not require caspase activity, as indicated by the lack of a significant effect of the pan-caspase inhibitor Z-VAD-fmk on cell death. In contrast, cyclosporine A pretreatment partly attenuated chloroquine-induced neuronal death under conditions of both basal and rapamycin-stimulated autophagy, thereby implicating the mitochondrial permeability transition pore in the caspase-independent cell death signaling process.

Cerebral ischemia induces ER stress, in part, via caspase-12 activation.^46, 47^ Recently, ER stress was found to be correlated with autophagic activation.^48, 49^ In yeast, ER stress was found to stimulate the assembly of the phagophore assembly site, induce autophagosome formation, and transport autophagosomes to vacuoles.^20, 49^ In a myocardial ischemia/reperfusion model, autophagy induction through therapeutic levels of ER stress inducers was found to prevent lethal injury.^48, 49^ Following ischemia, damaged protein aggregates and organelles accumulate due to defects in autophagy. The accumulation of these potentially toxic protein aggregates on organelle membranes can result in further organelle damage and ultimately neuronal death.^24, 50^ Thus, autophagy is thought to play a neuroprotective role in cerebral ischemia. ER stress induces ERAD II (ER-associated degradation II; autophagy/lysosome pathway), which then upregulates the molecular chaperones required for the degradation of misfolded and/or unfolded proteins in the ER lumen. By this mechanism, autophagy induction soon after HI would be expected to prevent more severe ER stress and protein aggregation. Because autophagy is both induced by and a reliever of ER stress,^51^ we hypothesized that impaired autophagy may further increase ER stress after neonatal HI. Moreover, because ER stress causes translational arrest,^52^ we expected that the levels of lysosomal enzymes such as cathepsin D, cathepsin B, and LAMP2 would be low after HI, possibly reflecting an ER stress-mediated decrease in protein translation. Our data showing an early increase in cathepsin D, cathepsin B and LAMP2 followed by decreases at 24 h, when increased caspase-12 expression colocalized with Beclin-1, confirmed this expectation. Translational arrest may result in a deleterious positive feedback cycle between impaired autophagy and ER stress after neonatal HI.

At 1 d after HI, LC3 had accumulated in activated microglia. Defective autophagy may contribute to inflammation via the NF-*κ*B pathway in cancer and other diseases.^53^ Specifically, p62 directly stimulates the NF-*κ*B pathway by interacting with TRAF6.^54^ In M2 macrophages, autophagy selectively degrades NF-*κ*B RELA/p65, and thus reduces the production of proinflammatory cytokines.^55^ In contrast, impaired autophagosome clearance and the consequent p62 accumulation might lead to the induction of proinflammatory responses. Consistent with this possibility, autophagy induction by GSK3B inhibitors was found to reduce neuroinflammation after brain ischemia.^56^ In our study, the number of autophagosomes was increased soon (1.5 h) after neonatal HI, when there was no p62 and ubiquitin accumulation. This suggested that the autophagic flux was intact at this time point. The death of the affected neurons and increased lysosomal activity in activated microglia after HI may have helped maintain the autophagic flux in this cell type. It is also possible that other autophagic pathways, such as chaperone-mediated autophagy, initially contributed to p62 clearance. This possibility is supported by the observed early increase in LAMP2 levels, as this protein is involved in chaperone-mediated autophagy.

Ubiquitinated cargo largely and specifically accumulates in neurons targeted for delayed cellular death after transient ischemia.^57^ In the present study, we attempted to determine whether protein aggregation after brain HI was attributable to impaired autophagy. We found that autophagic activity was increased in the early stages after HI, but then decreased at later time points. Owing to this, marked accumulation of protein ubiquinated aggregates p62 was delayed until 1 d after HI. It is known that protein aggregation on subcellular organelle membranes can cause multiple organelle failure and ultimately delayed neuronal death after neonatal HI.^12^ On the basis of our findings, we consider that early interventions to reduce autophagosome accumulation via restoration of lysosomal function or reduction of autophagosome synthesis and to activate other autophagic pathways may be beneficial after HI. Furthermore, we predict that such interventions may both limit the extent of neuronal death and ameliorate neuroinflammation. Conversely, in the early stages after HI when the autophagic flux is still intact, the induction of autophagy may have a neuroprotective role.

## Materials and methods

### Animal preparation

All procedures were approved by the Animal Care and Use Committee at Johns Hopkins University and complied with the United States Public Health Service Policy on the Humane Care and Use of Laboratory Animals and the Guide for the Care and Use of Laboratory Animals.^16^ Animal care was in accordance with the National Institutes of Health Guidelines and ensured animal comfort. Male piglets (weight, 1.5–2.5 kg each; age, 3–5 d) underwent sham surgery or HI induction. Anesthesia was induced with 5% isoflurane and a 50%:50% mixture of nitrous oxide and oxygen, and maintained with 2% isoflurane and a 70%:30% mixture of nitrous oxide and oxygen. The animals were intubated and mechanically ventilated, and then catheters were inserted into their femoral artery and vein. Fentanyl was administered (20 *μ*g/kg, iv) after venous catheter placement, and isoflurane was discontinued. The catheter placement took ~15 min. We administered vecuronium (0.2 mg/kg/h, iv) to all piglets to prevent ventilatory efforts during the HI exposure. The piglets received a continuous infusion of 5% dextrose in 0.45% saline (10 ml/h, iv).

### HI

The severity of HI in piglets can be varied by altering the hypoxia duration. Durations of 30–45 min have been used previously.^14, 15, 58, 59^ In the current study, we used a severe HI model, with 45 min of hypoxia. We induced whole-body hypoxia by decreasing the inspired oxygen concentration to 10% for 45 min to achieve an oxyhemoglobin saturation of 30–35%. We then administered 21% inspired oxygen to the piglets for 5 min. This transient reoxygenation was needed for cardiac resuscitation. Next, we occluded the endotracheal tube for 7 min to produce complete asphyxia. After this, the piglets were resuscitated with 50% inspired oxygen, manual chest compressions, and, if necessary, epinephrine (100 *μ*g/kg, iv).^14, 16^ Piglets that failed to show return of spontaneous circulation within 3 min were excluded from the study. After resuscitation, the vecuronium infusion was stopped and the concentration of inspired oxygen was decreased to 30%. Sodium bicarbonate was used to treat metabolic acidosis. Sham-operated animals underwent the same anesthesia protocol, but received 30% oxygen throughout the protocol without any HI. Rectal temperature was maintained at 38.5 °C (normal for piglets) with heating blankets and heating lamps throughout the duration of anesthesia in all groups. For piglets survived longer than 6 h of recovery, anesthesia was discontinued at 3 h and the piglets were returned to their home cage with littermates and were fed swine formula milk.

### Cresyl violet staining

Slide-mounted brain sections were prepared in 95% ethyl alcohol for at least 5 h at room temperature and then rinsed in 75% ethyl alcohol for 5 min and in distilled water for 5 min. Next, the sections were stained with 0.1% cresyl violet (Sigma, St. Louis, MO, USA) for 2–3 min and then rinsed quickly with distilled water. After decolorization in 75% ethyl alcohol for a few seconds, the sections were dehydrated in 95 and 100% ethyl alcohol for 2–3 min, cleared in xylene for 2–3 min, and mounted with Permount (Fisher Scientific Inc., Waltham, MA, USA) in a fume hood.

### Immunohistochemistry

At 6 h or 1 d after HI, the piglets were deeply anesthetized with 50 mg/kg pentobarbital sodium and transcardially perfused first with cold phosphate-buffered saline (PBS) and then with 4% paraformaldehyde (pH, 7.4). Their brains were removed, post-fixed in paraformaldehyde for 1 d, and protected in 30% sucrose. Next, frozen sections (20 *μ*m) were prepared and mounted on glass slides. For immunofluorescence analysis, the sections were blocked with 5% goat serum (Millipore, Bedford, MA, USA, S26-LITER) or donkey serum (Sigma, D9663) in 1 × PBS (Quality Biological Inc., Gaithersburg, MD, USA, 119-069-101) containing 0.25% Triton X-100 (Sigma, X100). The sections were then incubated overnight with the primary antibodies and subsequently with the secondary antibodies for 2 h at room temperature. The nuclei of the cells were stained with DAPI. We used the following primary antibodies: LC3 (1:200; Novus, Littleton, CO, USA, NB100-2220), NeuN (1:500; Millipore, MAB377), SQSTM1/p62 (1:200; Progen, Heidelberg, Germany, GP62-C), GFAP (1:1000; Dako, Santa Clara, CA, USA, Z0334), IBA1 (1:1000; Wako, Richmond, VA, USA, 019-19741), ubiquitin (1:200; Cell Signaling Technology, Danvers, MA, USA, 3936), cathepsin D (1:100; Santa Cruz Biotechnology, Dallas, TX, USA, sc-6486), AIF (1:250; Cell Signaling Technology, 5318), cleaved caspase-9 (1:200; Cell Signaling Technology, 9509), caspase-12 (1:200; Cell Signaling Technology, 2202), MLKL (1:100; Santa Cruz Biotechnology, sc-165025), PGAM5 (1:100; Abcam, Cambridge, MA, USA, ab126534), and RIP3 (1:100; Santa Cruz Biotechnology, sc-374639). All secondary antibodies used in this study were purchased from Invitrogen (Grand Island, NY, USA): Alexa Fluor-546 goat anti-mouse (A11030), Alexa Fluor-488 goat anti-rabbit (A11034), Alexa Fluor-633 goat anti-mouse (A21052), and Alexa Fluor-546 donkey anti-goat (A11056) antibodies.

### Image analysis

Microscopic images were acquired using a fluorescence Nikon Ti-E inverted microscope. The images were captured at 20 × and 60 × magnification, and at emission wavelengths of 620 nm (Alexa Fluor-546), 535 nm (Alexa Fluor-488), and 460 nm (DAPI). Exposure times were kept constant for all sections in each experiment. All 60 × images were acquired as z-stacks and focused using the Extended Depth of Focus module of Elements software (Nikon, Japan). All images were background-subtracted using the Elements software. Quantitative analyses were also performed using Elements: nuclei were detected using the Spot Detection algorithm; cells positive for LC3 or any other immunofluorescent marker were identified using the Detect Regional Maxima algorithm followed by global thresholding. The number of positive cells was normalized to the total number of cells observed. Intracellular puncta were detected using Spot Detection and normalized to the total number of cells. All quantifications were performed for a minimum of 1000–2000 cortical cells/piglet in each experiment.

### Western blot analysis

At 1.5 h, 3 h, 6 h, 1 d, or 2 d after HI or sham surgery, piglets were deeply anesthetized and killed by transcardial perfusion with cold PBS, and their brains were rapidly harvested. Tissue samples were collected from the fresh brain slabs by using micropunches. Frozen samples of the sensorimotor cortex were treated with ice-cold 1 × RIPA buffer (Cell Signaling Technology) and protease inhibitor cocktail (Invitrogen, Grand Island, NY, USA) at a weight-to-volume ratio of 0.1 g/1 ml. The tissue samples were homogenized with a hand-held electric homogenizer, and the homogenates were placed on ice to let the macroscopic tissue debris settle. They were then homogenized again and subsequently sonicated for 60 s. Finally, the samples were centrifuged at 20 000 *g* for 20 min at 4 °C. The supernatant was removed, and the protein concentration was determined using the Pierce BCA protein assay kit (Thermo Scientific, Rockford, IL, USA). The protein samples were denatured in a loading buffer (4 × LDS sample buffer, Invitrogen), separated using sodium dodecyl sulfate-polyacrylamide gel electrophoresis on 4–12% Tris-glycine gels, and then transferred to nitrocellulose membranes. The membranes were blocked in 3% non-fat milk at room temperature and then incubated overnight at 4 °C with the following primary antibodies: Beclin-1 (1:1000; Santa Cruz Biotechnology, sc-11427), LC3 (1:1000; Novus, NB100-2220), PIK3C3/VPS34 (1:1000; Invitrogen, 382100), SQSTM1 (1:1000; BD Bioscience, 610832), cathepsin D (1:1000; Santa Cruz Biotechnology, sc-6486), cathepsin B (1:1000, Abcam, ab58802), ubiquitin (1:1000; Cell Signaling Technology, 3936), LAMP2 (1:1000, Abcam, ab25631), spectrin (1:5000; Enzo Life Science International, BMLFG6090), phospho-ULK1 (1:1000; Cell Signaling Technology, 5869), ATG5 (1:1000; Sigma, A0731), PARP (1:200; Cell Signaling Technology), cleaved caspase-3 (1:200; Cell Signaling Technology), PGAM5 (1:100; Abcam, ab126534), MLKL (1:100; Santa Cruz Biotechnology, sc-165025), RIP3 (1:100; Santa Cruz Biotechnology, sc-374639), and monoclonal mouse anti-*β*-actin IgG (1:3000, Santa Cruz Biotechnology). The membranes were rinsed before being incubated with the corresponding secondary antibodies (anti-rabbit IgG, anti-goat IgG, Thermo Scientific; anti-mouse IgG, General Electric Healthcare Life Sciences, Pittsburgh, PA, USA) for 1 h at room temperature. *β*-Actin was the protein loading control. Finally, the membranes were washed thrice, and their immunoreactivity was assessed using enhanced chemiluminescence (Thermo Scientific) via the quantification of optical density. Immunoreactive band intensities were determined using the ImageJ software (National Institutes of Health, Bethesda, MD, USA). The proteins obtained from two piglets per group (1.5, 3, 6, and 1 d) were run on a single gel in three separate experiments.

### Subcellular fractionation

Brains perfused with cold PBS were removed at 1 d after HI, homogenized in buffered ice-cold sucrose solution containing 0.32 M sucrose (Fisher Scientific, BP220-212), 10 mM HEPES (HyClone, Logan, UT, USA, SH30237.01), and protease and phosphatase inhibitors. The homogenates were then centrifuged at 800 *g* for 10 min at 4 °C to spin the nuclei down. The resultant supernatant was collected and centrifuged again at 20 000 *g* for 20 min at 4 °C to spin down the crude lysosomal fractions. The supernatant thus obtained was further centrifuged at 100 000 *g* for 1 h at 4 °C to spin down the crude membrane fraction. The pellets obtained at each centrifugation step were resuspended in the homogenization buffer. Both the supernatants and the suspended pellets were centrifuged again to minimize cross contamination from different subcellular fractions. Finally, all pellet fractions were resuspended in homogenization buffer and analyzed using protein gel blots.

### Real-time PCR

Total RNA was isolated from the cortex by using Trizol (Invitrogen, 15596-018) and then reverse transcribed into cDNA by using the Verso cDNA kit (Thermo Scientific, AB1453B) according to the manufacturer’s instructions. The cDNA TaqMan Universal Master Mix II (Applied Biosystems, Foster City, CA, USA, 4440040,) was used for quantitative real-time PCR amplification. In brief, the reactions were performed in duplicate by mixing 2 × TaqMan Universal Master Mix II, 1 ml cDNA (equivalent to 50 ng RNA/reaction), and 20 × TaqMan Gene Expression Assay solution to obtain a final volume of 20 ml. We performed TaqMan Gene Expression assays for the following genes: LC3B (Mm00782868_sH), Beclin-1 (Mm01265461_m1), Atg12 (Mm00503201_m1), cathepsin D (Mm00515586_m1), SQSTM1/p62 (Mm00448091_m1), and GAPDH (Mm99999915_g1; all from Applied Biosystems). The reactions were amplified and quantified using a 7900HT Fast Real-Time PCR System and its software (Applied Biosystems). The PCR protocol consisted of 1 cycle at 50 °C for 2 min and at 95 °C for 10 min, followed by 40 cycles at 95 °C for 15 s and at 60 °C for 1 min. Gene expression levels were normalized to the GAPDH expression level. Relative mRNA amounts were calculated using the comparative Ct method.^55^

### Cathepsin D assay

We used a fluorometric cathepsin D assay kit from Abcam (ab65302) according to the manufacturer’s instructions. In brief, the piglets were anesthetized, perfused with ice-cold saline, and decapitated. The cortical tissue surrounding the injury site (diameter, 5 mm) was dissected and homogenized in ice-cold cell lysis buffer provided in the assay kit. The homogenates were centrifuged at 15 000 *g* for 5 min at 4 °C. Protein concentration of the supernatant was estimated by the BCA method. Then, 50 ng protein was incubated with the cathepsin D substrate mixture at 37 °C for 1 h. The fluorescence released from the synthetic substrate by tissue cathepsin D was estimated using a fluorescence plate reader (Synergy Hybrid, Biotek, Winooski, VT, USA) at excitation and emission wavelengths of 328 and 460 nm, respectively.

### Embryonic cortical neuronal culture

Primary cortical neuronal cultures were prepared from embryonic day 16 C57Bl/6 mice as described previously.^60^ Primary cortical neurons were grown in a culture medium composed of Neurobasal medium (Invitrogen, Carlsbad, CA, USA), 2% B27 supplement (Invitrogen), 2 mM l-glutamine, and 1% penicillin-streptomycin, as described previously.^60^ After 3 days of culture, a third of the medium was replaced with fresh, L-glutamine-free medium containing 5 *μ*M cytosine arabinofuranoside (Sigma) to arrest non-neuronal cell growth. Experiments were conducted on the 12th day of culture, by which time the cultures consisted primarily of neurons (>95% MAP-2–immunoreactive cells; MAP-2 was obtained from Chemicon (Temecula, CA, USA).

### Assessment of cell viability/toxicity

Primary cortical neurons were treated with rapamycin (5 *μ*mol/l for 1 d) or vehicle control *in vitro* and then nutrient deprived for 1 d in the absence and presence of chloroquine (10 *μ*mol/l for 1 d). Another sample of primary cortical neurons was pretreated with chloroquine (10 *μ*mol/l) with or without treatment with rapamycin (5 *μ*mol/l) for 1 d in the presence of Z-VAD-fmk (20 *μ*mol/l), cyclosporine A (20 *μ*mol/l), or dimethyl sulfoxide (control). Cell viability was determined using a quantitative colorimetric MTT assay as described previously,^61, 62^ and the results were expressed as the percentage of viable cells in the control cultures.

### Statistical analysis

Statistical analyses were performed using Sigma Plot software (version 12) and GraphPad Prism (version 4). One-way analysis of variance was followed by a post-hoc test (Bonferroni, Tukey, or SNK *t*-tests) for parametric data (normality and equal variance passed). Kruskal–Wallis analysis of ranks followed by the Dunn post-hoc test was used for non-parametric data (normality and/or equal variance failed). The two-tailed Mann–Whitney rank sum test (non-parametric data) or the two-tailed unpaired Student *t*-test was used to compare results between two groups. *P*-values of ≤0.05 were considered significant.
