# Supplementary material for: Impaired autophagosome clearance contributes to neuronal death in a piglet model of neonatal hypoxic-ischemic encephalopathy
Source: Cell Death Dis. 2017 Jul 13;8(7):e2919–. doi: 10.1038/cddis.2017.318 (PMC5550864; doi:10.1038/cddis.2017.318)
Supplement: Supplementary Figure Legends [file cddis2017318x1.docx]

**Supplement figures legends**

**Supplementary Figure S1.** Early decreases in phosphorylation of mammalian target of rapamycin (mTOR) and p70S6K are consistent with enhanced autophagic signaling. (A) Western blots of mTOR and p70S6K in the cortical tissue lysates from sham-operated and HI piglets at the indicated time points. Each lane corresponds to an individual animal (2 per time point). (B, C) Densitometric analysis of (B) mTOR and (C) p70S6K data from (A) normalized to the loading control, β-actin. Data are presented as mean ± SD (n = 4, **P* < 0.05, ***P* < 0.01 vs. the sham-operated group).

**Supplementary Figure S2.** Autophagosome accumulation is present at 1 d of recovery in multiple brain regions that are selectively vulnerable to HI. Images of brain sections stained with antibodies against the autophagy marker LC3 and the neuronal marker NeuN in the regions of sensorimotor cortex, putamen, thalamus, subcortical white matter, and CA1 hippocampus. Scale bar = 50µm.

**Supplementary Figure S3.** Autophagy impairment after HI is associated with neuronal apoptotic cell death signaling. (A) Western blot analysis of spectrin breakdown products (150 kDa and 120 kDa), cleaved caspase-3 (19 kDa), and cleaved PARP breakdown products (89 kDa) in cortical tissues from sham-operated and HI piglets. (B–D) Corresponding densitometric analysis of (B) spectrin, (C) cleaved caspase-3, and (D) cleaved PARP bands with respect to β-actin (n = 4, ***P* < 0.01).

**Supplementary Figure S4.** Autophagy impairment after HI is associated with multiple cell death signaling pathways. (A–F) Images of cortical brain sections from sham-operated and HI piglets stained for apoptotic cell death markers (A) cleaved caspase-9, (C) AIF, and (E) caspase-12. Scale bar = 10 µm. Corresponding quantification of cells that were single-positive for the indicated cell death associated signaling molecules and double-positive for Beclin-1plus (B) cleaved caspase-9 (***P* < 0.01), (D) AIF (***P* < 0.01), and (F) caspase-12 (***P* < 0.01). The percentages of double-positive versus single-positive cells are indicated (n = 3; data are represented as mean ± SD).

**Supplementary Figure S5.** Autophagy impairment after HI co-localizes with downstream RIP1-dependent necrosis signaling proteins. (A–F) Images of cortical brain sections from sham-operated and HI piglets stained for programmed necrosis markers: (A) RIP3, (C) MLKL, and (E) PGAM5. Scale bar = 10 µm. Corresponding quantification of cells single-positive for the indicated necrosis markers and double-positive for Beclin-1 plus (B) RIP3 (***P* < 0.01), (D) MLKL (***P* < 0.01), and (F) PGAM5 (***P* < 0.01). The percentages of double-positive versus single-positive cells are indicated (n = 3; data are presented as mean ± SD).
