# Supplementary figures and images for: Impaired autophagosome clearance contributes to neuronal death in a piglet model of neonatal hypoxic-ischemic encephalopathy
Source: Cell Death Dis. 2017 Jul 13;8(7):e2919–. doi: 10.1038/cddis.2017.318 (PMC5550864; doi:10.1038/cddis.2017.318)

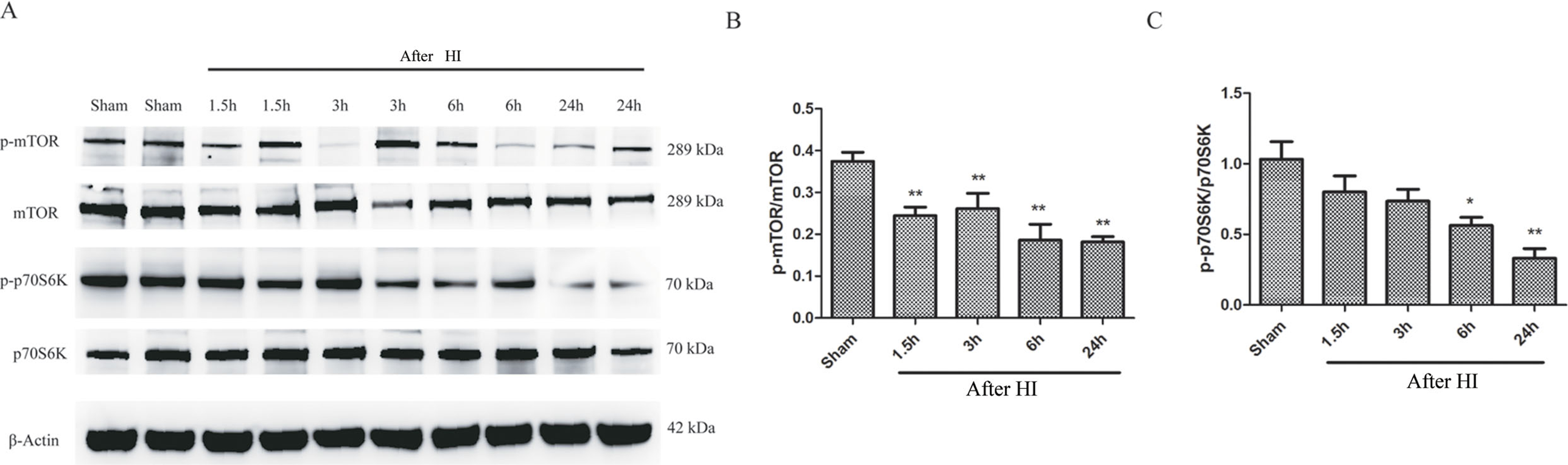

Supplement: Supplementary Figure S1 [file cddis2017318x2.tif]

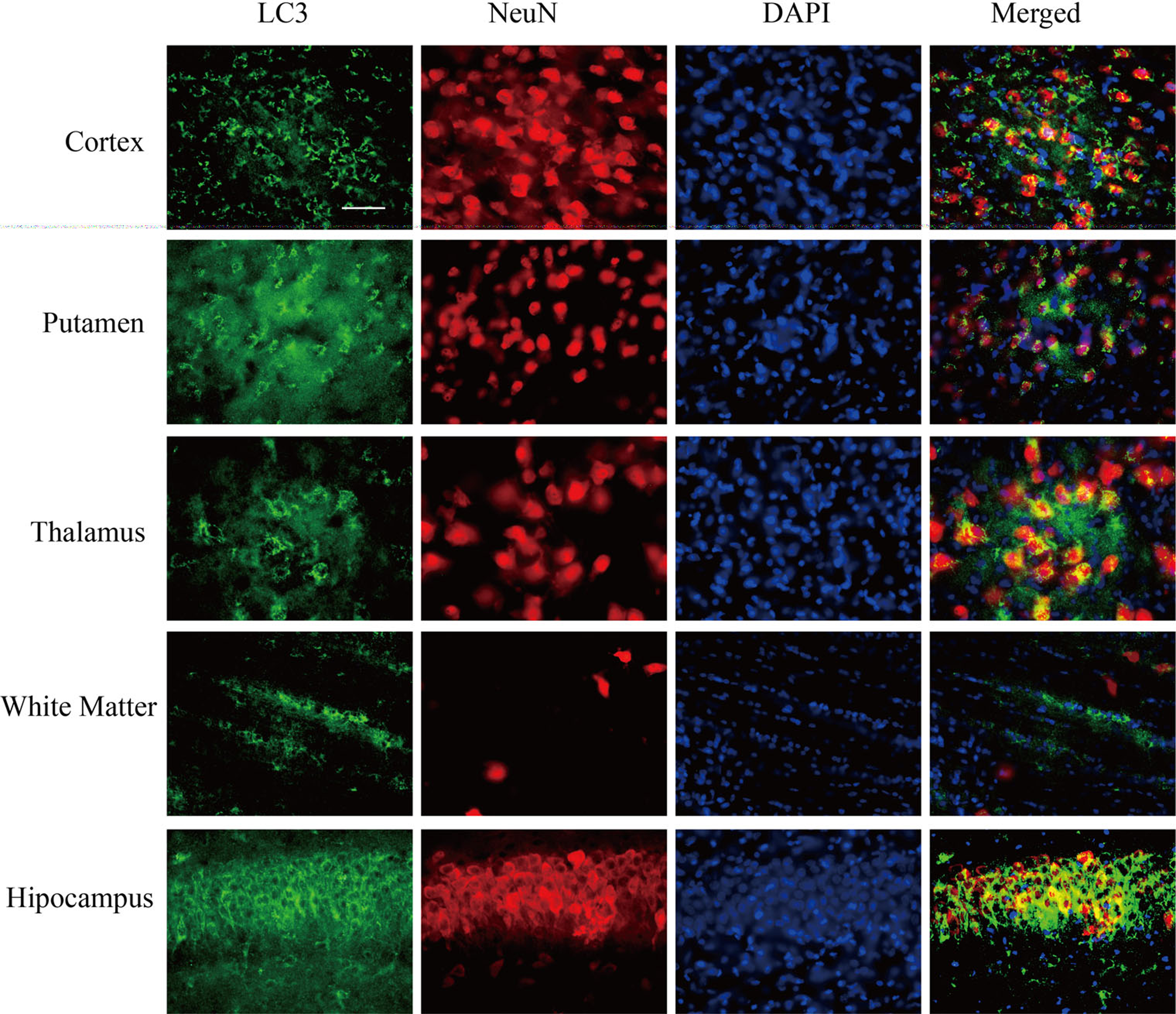

Supplement: Supplementary Figure S2 [file cddis2017318x3.tif]
